# Dual-Channel Cosine Function Based ITD Estimation for Robust Speech Separation

**DOI:** 10.3390/s17061447

**Published:** 2017-06-20

**Authors:** Xuliang Li, Zhaogui Ding, Weifeng Li, Qingmin Liao

**Affiliations:** Department of Electronic Engineering/Graduate School at Shenzhen, Tsinghua University, Beijing 100084, China; 64795992@163.com (X.L.); dingzhaogui@126.com (Z.D.); liaoqm@tsinghua.edu.cn (Q.L.)

**Keywords:** delay-and-sum beamforming, binary time-frequency mask, cosine function

## Abstract

In speech separation tasks, many separation methods have the limitation that the microphones are closely spaced, which means that these methods are unprevailing for phase wrap-around. In this paper, we present a novel speech separation scheme by using two microphones that does not have this restriction. The technique utilizes the estimation of interaural time difference (ITD) statistics and binary time-frequency mask for the separation of mixed speech sources. The novelties of the paper consist in: (1) the extended application of delay-and-sum beamforming (DSB) and cosine function for ITD calculation; and (2) the clarification of the connection between ideal binary mask and DSB amplitude ratio. Our objective quality evaluation experiments demonstrate the effectiveness of the proposed method.

## 1. Introduction

A common example of the well-known ‘cocktail party’ problem is the situation in which the voices of two speakers overlap. How to solve the ‘cocktail party’ problem and obtain an enhanced voice of a particular speaker in machines have grabbed serious attention of researchers.

As for single-channel speech separations, independent component analysis (ICA) [1] and nonnegative-matrix factorization (NMF) [2] are the conventional methods. However, the assumption that signals are statistically independent in ICA and the model in NMF is linear limit their applications. Moreover, NMF generally requires a large amount of computation to determine the speaker independent basis. Recently, in [3], the authors proposed an online adaptive process independent of parameter initialization, with noise reduction as a pre-processing step. Using adaptive parameters computed frame-by-frame, this article constructs a Time Frequency (TF) mask for the separation process. In [4], the authors proposed a pseudo-stereo mixture model by reformulating the binaural blind speech separation algorithm for the monaural speech separation problem. The algorithm estimates the source characteristics and constructs the masks with the parameters estimated through a weighted complex 2D histogram.

Normally, multiple channel sources are separated by measuring the differences of arrival time and sound intensity between microphones [5,6], which are also referred to as the interaural time differences (ITD) and the interaural intensity differences (IID). Interaural phase differences (IPD) have been used in [7,8]. The authors proposed a speech enhancement algorithm that utilizes phase-error based filters that depend only on the phase of the signals. Performances of the above systems depend on how the ITD (or IPD) threshold is selected. Instead of a fixed threshold, in [9], the authors employed a statistical modeling of angle distributions together with a channel weighting to determine which signal components belong to the target signal and which components are part of the background. In [10], the authors proposed a method based on a prediction of the coherence function and then estimated the signal to noise ratio (SNR) to generate Wiener filter. In [11], the author presented a method based on independent component analysis (ICA) and binary time-frequency masking. In [12], the authors proposed that a rough estimate of channel level difference (CLD) threshold yielding the best Signal-to-Distortion Ratio (SDR) could be obtained by cross-correlating the separated sounds. In addition, a combination of negative matrix factorization (NMF) with spatial localization via the generalized cross correlation (GCC) is applied for two-channel speech separation in [13]. For two-channel convolutive source separation, as the number of parameters in the NMF2D grows exponentially and the number of frequency basis increases linearly, the issues of model-order fitness, initialization and parameters estimation become even more critical. In [14], the authors proposed a Gaussian Expectation Maximization and Multiplicative Update (GEM-MU) algorithm to calculate the NMF2D with adaptive sparsity model and to utilize a Gamma-Exponential process in order to estimate the number of components and number of convolutive parameters in NMF2D.

**Figure 1:**
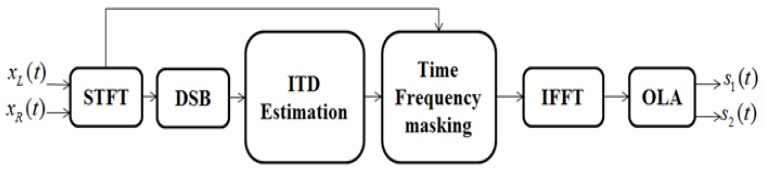
Block diagram of the proposed approach. STFT: Short Time Fourier Transform, DSB: Delay-and-Sum Beamforming, ITD: Interaural Time Difference, IFFT: Inverse Fast Fourier Transform, OLA: OverLapping and Adding.

The goal of this paper is to cope with competing-talker scenarios by dual-channel mixtures. In this study, we use DSB to generate the cosine function that evaluates ITD by using several frames of the short-time Fourier transform (STFT) and makes target and competing signals have the same characteristics. Then, we utilize the binary time-frequency mask to obtain the target source. There are two contributions in this paper:

(1) we novelly upgrade delay-and-sum beamforming (DSB) [15] for estimating the ITD; and
(2) for the first time, we clarify the connections between ideal binary mask and DSB amplitude ratio. The framework of our approach is illustrated in Figure 1. Moreover, our proposed algorithm can handle the problem of phase wrap-around.

The remainder of this paper is organized as follows: Section 2 provides an overview of time difference model. Our proposed approach including system overview and algorithm will be discussed in Section 3. In Section 4, we will introduce source separation. Then, Section 5 shows our evaluations of the system. Finally, Section 6 puts forward the main conclusions of the work.

## 2. Time Difference Model

We suppose that there are *I* (I=2) sources (subscript 1 to represent the target and subscript 2 to represent the noise) in a sonic environment. The signals from two different microphones are defined, respectively, as:

(1) $$\begin{array}{cc} x_{L}\left(t\right) & =\sum_{i=1}^{I}a_{i}^{L}s_{i}\left(t\right),\\ x_{R}\left(t\right) & =\sum_{i=1}^{I}a_{i}^{R}s_{i}\left(t-\tau_{i}\right), \end{array}$$

where $a_{i}^{L}$ and $a_{i}^{R}$ denote the weighted coefficients of the recordings of the left and right microphone from the *i*-th source separately. $\tau_{i}$ is the time delay of arrival (TDOA) of the *i*-th source between two microphones. Equation (1) can be simplified as:

(2) $$\begin{array}{cc} x_{L}\left(t\right) & =\sum_{i=1}^{I}s_{i}\left(t\right),\\ x_{R}\left(t\right) & =\sum_{i=1}^{I}b_{i}s_{i}\left(t-\tau_{i}\right), \end{array}$$

where $b_{i}$ is the ratio of $a_{i}^{L}$ and $a_{i}^{R}$. By the short-time Fourier transform (STFT), the signals can be expressed as:

(3) $$\begin{array}{cc} X_{L}\left[m,k\right] & =\sum_{i=1}^{I}S_{i}\left[m,k\right],\\ X_{R}\left[m,k\right] & =\sum_{i=1}^{I}b_{i}S_{i}\left[m,k\right]\times e^{-j\omega_{k}\tau_{i}}, \end{array}$$

where *m* is the frame index and $\omega_{k}=2\pi k/K$. *k* and *K* are the frequency index and total window length, respectively. Under the assumption of Wdisjoint orthogonal [16], Equation (3) can be rewritten as:

(4) $$\begin{array}{cc} X_{L}\left[m,k\right] & \approx S_{i}\left[m,k\right],\\ X_{R}\left[m,k\right] & \approx b_{i}S_{i}\left[m,k\right]\times e^{-j\omega_{k}\tau_{i}}. \end{array}$$

Thus, once the TDOA is obtained, we can make a simple binary decision concerning whether the time-frequency bin [m,k] is likely to belong to the target speaker or not.

## 3. Proposed Approach

Delay-and-sum (DSB) is an effective means for speech enhancement. Our method is based on DSB under the anechoic condition in the time-frequency domain. In DSB, the enhanced speeches in the time-frequency domain are modeled as:

(5) $$\begin{array}{cc} Y_{1}\left[m,k\right]= & \frac{X_{L}\left[m,k\right]+X_{R}\left[m,k\right]\times e^{j\omega_{k}{\hat{\tau }}_{1}}}{2},\\ Y_{2}\left[m,k\right]= & \frac{X_{L}\left[m,k\right]+X_{R}\left[m,k\right]\times e^{j\omega_{k}{\hat{\tau }}_{2}}}{2}, \end{array}$$

where $Y_{1}\left[m,k\right]$ and $Y_{2}\left[m,k\right]$ are the enhanced speech of target and interferer, respectively.

Theoretically, once the correct estimations of $\tau_{1}$ and $\tau_{2}$ are obtained, Equation (5) is written as:

(6) $$\begin{array}{c} \frac{Y_{1}\left[m,k\right]}{Y_{2}\left[m,k\right]}=\left\{\begin{array}{cc} \frac{1+b_{1}}{1+b_{1}\times e^{j\omega_{k}\left(\tau_{2}-\tau_{1}\right)}}, & \mathrm{if}\;\left[m,k\right]\;\in \;s_{1},\\ \frac{1+b_{2}\times e^{j\omega_{k}\left(\tau_{1}-\tau_{2}\right)}}{1+b_{2}}, & \mathrm{if}\;\left[m,k\right]\;\in \;s_{2}. \end{array}\right. \end{array}$$

We define g[k] as:

(7) $$g\left[k\right]=\frac{1}{M}\sum_{m=1}^{M}{\left|\frac{Y_{1}\left[m,k\right]}{Y_{2}\left[m,k\right]}\right|}^{\mathrm{sgn}(\left|1-\frac{Y_{1}\left[m,k\right]}{Y_{2}\left[m,k\right]}\right|)},$$

where

(8) $$\mathrm{sgn}(x)=\left\{\begin{array}{cc} 1, & x\geq 0,\\ -1, & x<0. \end{array}\right.$$

According to Equations (6) and (7), we treat $g_{The}\left[k\right]$ as the theoretical result of g[k]. Under the assumption of far-field ($b_{1}$ ≈ $b_{2}$), $g_{The}\left[k\right]$ is simplified to

(9) $$\begin{array}{c} g_{The}\left[k\right]\approx \left|\frac{1+b_{1}\times e^{j\omega_{k}\left(\tau_{2}-\tau_{1}\right)}}{1+b_{1}}\right|. \end{array}$$

We may obtain

(10) $$\begin{array}{c} g_{The}\left[k\right]\approx \sqrt{1-\frac{2b_{1}\left(1-\cos\left(\omega_{k}\times \left(\tau_{2}-\tau_{1}\right)\right)\right)}{{\left(1+b_{1}\right)}^{2}}}, \end{array}$$

where $g_{The}\left[k\right]$ is the cosine function. Specially, if $b_{1}$ equals 1, we have

(11) $$\begin{array}{c} g_{The}\left[k\right]\approx \left|\cos\left(\frac{\omega_{k}\times \left(\tau_{2}-\tau_{1}\right)}{2}\right)\right|. \end{array}$$

Obviously, the maximum of $g_{The}\left[k\right]$ is 1. Furthermore, we let $g_{real}\left[k\right]$ be the real data of g[k] according to Equation (6). To ensure that the maximum of $g_{real}\left[k\right]$ is 1, we rectify $g_{real}\left[k\right]$ as:

(12) $$\begin{array}{c} g_{real\_r}\left[k\right]=g_{real}\left[k\right]+1-\max g_{real}\left[k\right]. \end{array}$$

We define the minimum of $g_{real}\left[k\right]$ as $g_{min}\left[k\right]$. Under the correct estimations of $\tau_{1}$ and $\tau_{2}$, $g_{real}\left[k\right]$ approximately equals $g_{The}\left[k\right]$. According to Equation (10), $b_{1}$ can be estimated as:

(13) $$\begin{array}{c} {\hat{b}}_{1}=\frac{1-g_{min}\left[k\right]}{1+g_{min}\left[k\right]}. \end{array}$$

**Figure 2:**
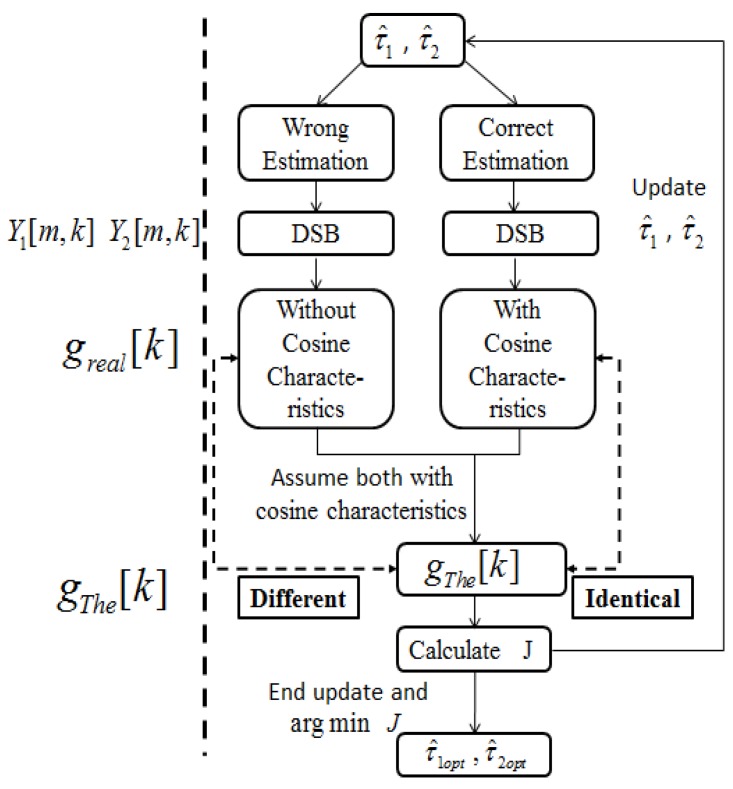
Float chart of ITD estimation. ${\hat{\tau }}_{1}$ and ${\hat{\tau }}_{2}$ are the estimation values of $\tau_{1}$ and $\tau_{2}$. If correct estimations of $\tau_{1}$ and $\tau_{2}$ are obtained, the cosine characteristics of $g_{The}\left[k\right]$ is identical to $g_{real}\left[k\right]$. In spite of the fact that there would be no cosine characteristics in $g_{real}\left[k\right]$ based on incorrect estimation results, we can still follow the cosine characteristics to calculate $g_{The}\left[k\right]$. Obviously, $g_{The}\left[k\right]$ is different to $g_{real}\left[k\right]$ in this situation. We find the true value of ${\hat{\tau }}_{1}$ and ${\hat{\tau }}_{2}$ iteratively. The ${\hat{\tau }}_{1}$ and ${\hat{\tau }}_{2}$ will be updated until $g_{The}\left[k\right]$ is identical to $g_{real}\left[k\right]$.

**Figure 3:**
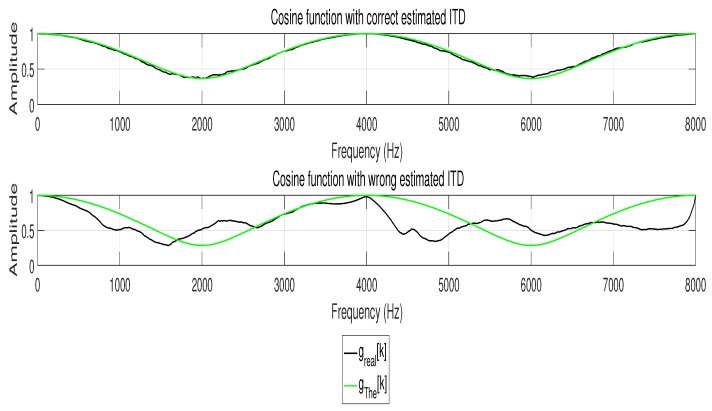
Cosine function with different ITD estimation. Obviously, $g_{The}\left[k\right]$ is identical to $g_{real\_r}\left[k\right]$ with correct ITD estimation, while $g_{The}\left[k\right]$ is different to $g_{real\_r}\left[k\right]$ with incorrect ITD estimation.

Figure 2 demonstrates the process of ITD estimation. Figure 3 gives an example about the cosine functions with different estimations of ITD.

We define the criterion function as:

(14) $$\begin{array}{c} J=\frac{1}{\Sigma_{k=1}^{K}\left|g_{real\_r}\left[k\right]-g_{The}\left[k\right]\right|}. \end{array}$$

Because of the periodicity of Trigonometric function, we fix $|\omega_{k}\left(\tau_{1}-\tau_{2}\right)|<\pi$. We use the summation on all frequency bands to avoid phase wrap-around problem. Then, we have

(15) $$\begin{array}{c} {\hat{\tau }}_{1_{opt}},{\hat{\tau }}_{2_{opt}}={\arg\max}_{{\hat{\tau }}_{1},{\hat{\tau }}_{2}}J. \end{array}$$

## 4. Source Separation

After obtaining the ITD and attenuation coefficients (namely $b_{1}$ and $b_{2}$), we adopt the masking method to separate the target and competing sources. Firstly, we illustrate the effects of attenuation coefficients. Then, we utilize the time-frequency mask based on the DSB ratio.

### 4.1. The Effects of Weighted Coefficients

In Equation (10), we assume $b_{1}$ ≈ $b_{2}$, but sometimes experiment settings can not meet this hypothesis strictly. In this section, we set different values of $b_{1}$ and $b_{2}$ artificially to demonstrate the effectiveness of the criterion function in Equation (14). We verify the effects of $b_{1}$ and $b_{2}$ with a simple example. Assume that

(16) $$\begin{array}{cc} x_{l}\left(t\right)= & s_{1}\left(t\right)+s_{2}\left(t\right),\\ x_{2}\left(t\right)= & b_{1}\times s_{1}\left(t-6.1\right)+b_{2}\times s_{2}\left(t-1.9\right). \end{array}$$

**Figure 4:**
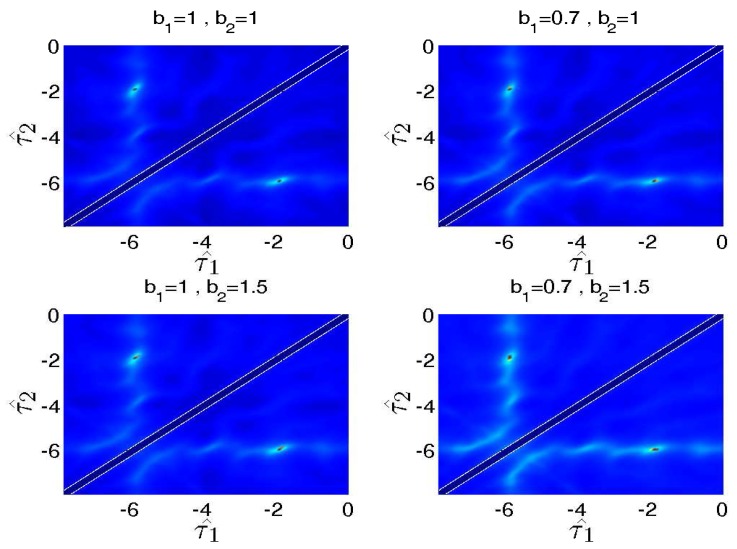
Source localization with different $b_{1}$ and $b_{2}$. The source localization are conducted in four different settings: (**1**) $b_{1}=1$, $b_{2}=1$; (**2**) $b_{1}=0.7$, $b_{2}=1$; (**3**) $b_{1}=1$, $b_{2}=1.5$; and (**4**) $b_{1}=0.7$, $b_{2}=1.5$. The ITD estimation is valid for all of the settings.

The details are shown in Figure 4. We can observe that even experiment settings do not meet the assumption that $b_{1}$ ≈ $b_{2}$ strictly, and the ITD still can be estimated accurately. Moreover, though the values of ${\hat{b}}_{1}$ and ${\hat{b}}_{2}$ are rough, the binary mask is free from attenuation coefficients since the DSB based mask only relies on ITD information.

### 4.2. Mask Based on DSB Ratio

Under the assumption of Wdisjoint orthogonal, the ideal ratio mask is defined using a priori energy ratio $R_{SNR}\left[m,k\right]$ [17]:

(17) $$\begin{array}{c} R_{SNR}\left[m,k\right]=\frac{{\left|Y_{1}\left[m,k\right]\right|}^{2}}{{\left|Y_{1}\left[m,k\right]\right|}^{2}+{\left|Y_{2}\left[m,k\right]\right|}^{2}}. \end{array}$$

In addition, the ideal binary is of the form:

(18) $$B[m,k]=\left\{\begin{array}{cc} 1, & R_{SNR}\left[m,k\right]\geq \lambda ,\\ 0, & R_{SNR}\left[m,k\right]<\lambda , \end{array}\right.$$

where λ is set to be a value in 0.2–0.8.

In our theoretical framework, $\left|\frac{1+b_{1}}{1+b_{1}\times e^{j\omega_{k}\left(\tau_{2}-\tau_{1}\right)}}\right|$ is greater than 1 according to Equation (6), while $\left|\frac{1+b_{2}\times e^{j\omega_{k}\left(\tau_{2}-\tau_{1}\right)}}{1+b_{2}}\right|$ is always less than 1. Then, the DSB ratio is of the form:

(19) $$\begin{array}{c} R_{DSB}\left[m,k\right]=\left\{\begin{array}{cc} |\frac{Y_{1}\left[m,k\right]}{Y_{2}\left[m,k\right]}|\geq 1, & \mathrm{if}\;\left[m,k\right]\in \;s_{1},\\ |\frac{Y_{1}\left[m,k\right]}{Y_{2}\left[m,k\right]}|<1, & \mathrm{if}\;\left[m,k\right]\in \;s_{2}. \end{array}\right. \end{array}$$

Comparing $R_{DSB}\left[m,k\right]$ to 1, the binary time-frequency mask is obtained as:

(20) $$\begin{array}{c} M\left[m,k\right]=\left\{\begin{array}{cc} 1, & \mathrm{if}\;R_{DSB}\left[m,k\right]\geq 1,\\ 0, & \mathrm{otherwise}. \end{array}\right. \end{array}$$

It is easy to find that when λ is set to 0.5, B[m,k] is equivalent to M[m,k]. Equations (6) and (20) demonstrate the essence that λ=0.5 provides the best performance under the assumption of Wdisjoint orthogonal. Then, the speech can be separated as:

(21) $$\begin{array}{cc} {\hat{S}}_{1}\left[m,k\right] & =M\left[m,k\right]X_{1}\left[m,k\right],\\ {\hat{S}}_{2}\left[m,k\right] & =\left(1-M\left[m,k\right]\right)X_{2}\left[m,k\right], \end{array}$$

where X[m,k] is defined as:

(22) $$\begin{array}{c} X_{i}\left[m,k\right]=\frac{1}{2}\left[\mathrm{DFT}\left(x_{L}\left(t\right)\right)+\mathrm{DFT}\left(x_{R}\left(t-t_{i}\right)\right)\right]. \end{array}$$

Finally, we can obtain the separated speech waveforms using the Inverse Fast Fourier Transform (IFFT) and OverLapping and Adding (OLA).

## 5. Experimental Evaluations

In this section, we first describe the experimental data and evaluation criteria that we used, and then present experimental results.

### 5.1. Experimental Setup

**Figure 5:**
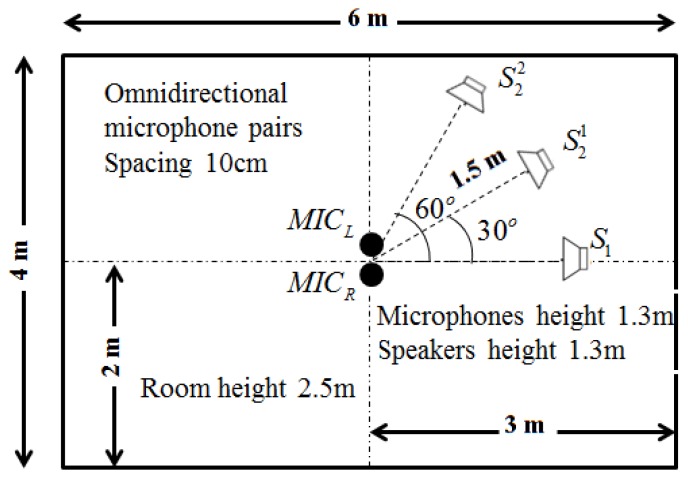
Placement of the microphones and sound sources. $S_{1}$ is the target source. $S_{2}^{1}$ and $S_{2}^{2}$ are the competing sources in two different environments, respectively.

Figure 5 depicts the simulated experimental set-up. The sources are selected from the TIMIT database [18]. The sample rate of these audio files is 16,000 Hz. For simulated data, we evaluate the target speech separation performance using Perceptual Evaluation of Speech Quality (PESQ), $C_{sig}$, $C_{bak}$ and $C_{ovl}$ [19]. These new composite measures show moderate advantages over the existing objective measures [19]. To meet the SiSEC 2010 campaign’s evaluation criteria, we adopt the standard Source-to-Interference Ratio (SIR) [20] for SiSEC 2010 test data. For these objective measures, the higher values mean better performance.

The window length is 1024 samples with an overlap of 75%. We can calculate the voiced frames detected by Voice Active Detector (VAD) [21] to avoid the situation that $Y_{2}\left[m,k\right]=0$. Actually, $Y_{2}\left[m,k\right]=0$ hardly occurs and we do not have this operation in our experiment. Once the amplitude of $Y_{2}\left[m,k\right]$ is nonzero, we treat $Y_{2}\left[m,k\right]$ as one of the speakers.

### 5.2. Simulated Data

**Figure 6:**
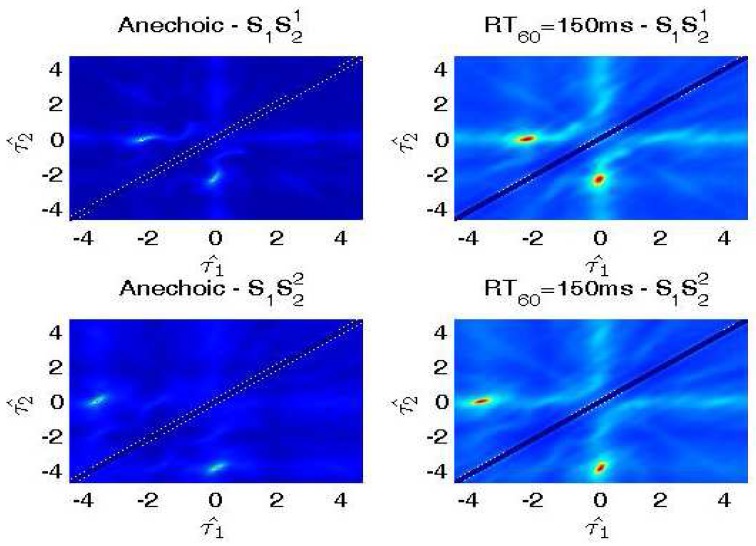
ITD estimation results in different environments. The horizontal coordinate corresponds to $\hat{\tau_{1}}$, and the vertical coordinate corresponds to $\hat{\tau_{2}}$. In fact, we can only process the lower triangular matrix because the estimations have symmetric properties.

**Figure 7:**
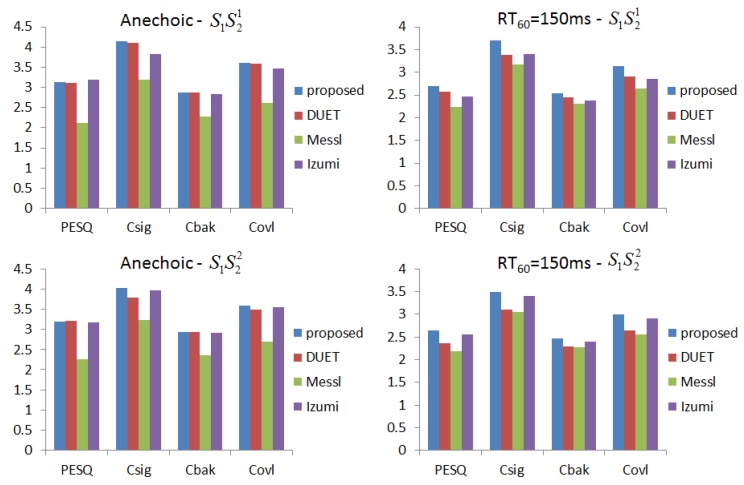
The target speech performance of different methods in terms of Perceptual Evaluation of Speech Quality (PESQ), $C_{sig}$, $C_{bak}$ and $C_{ovl}$.

**Figure 8:**
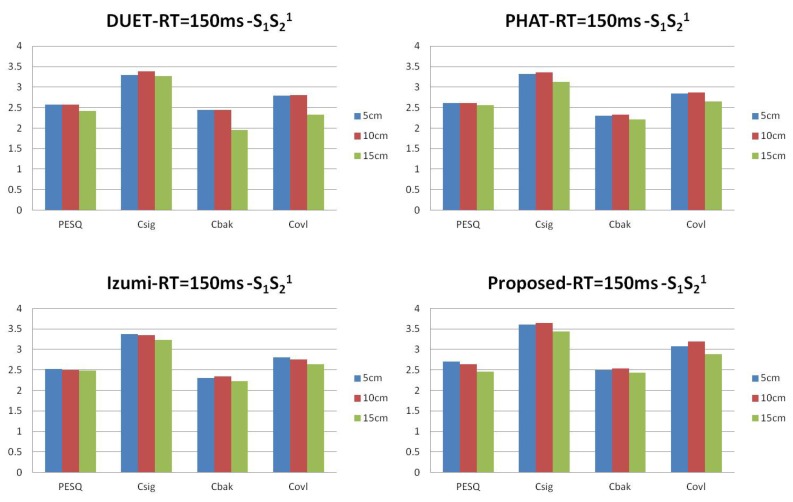
The target speech performance of different microphone distances in terms of Perceptual Evaluation of Speech Quality (PESQ), $C_{sig}$, $C_{bak}$ and $C_{ovl}$.

We generate data for the setup in Figure 5 with source signals of duration 2 s. Reverberation simulations are accomplished using the Room Impulse Response (RIR) open source software package [22] based on the image method. We generate 100 mixed sentences for each experimental set. Table 1 and Table 2 show the ITD estimated results in terms of mean square errors. In our experiment, the units of ITD are represented by τ×fs. We compare our approach with other existing DUET [23], Messl [24], and Izumi [25] methods. Unlike the algorithms based on coherence, our method consolidates the estimation of $\tau_{1}$ and $\tau_{2}$ into one cosine function. Our method acquires better ITD estimation. Table 3 shows the relations between microphone distances with ITD estimated results. The real ITD is proportional to the distances. The estimated ITDs calculated by our method meet this rule. For all of the distances in our experiment, the proposed method provides better ITD estimations that influence the separation results. Figure 6 shows the details with ITD estimation. Though our method does not take reverberation into consideration, the results demonstrate that our method is effective for low reverberation ($RT_{60}$ = 150 ms) conditions. Figure 7 shows the target source separation performance and illustrates that our method has comparable performance. Figure 8 shows the target source separation performance for different microphone distances. For different microphone distances, the source separation performances are effective. Compared with other methods, the proposed method yields better results for all of the microphone distances.

### 5.3. SiSEC 2010 Test Data

**Figure 9:**
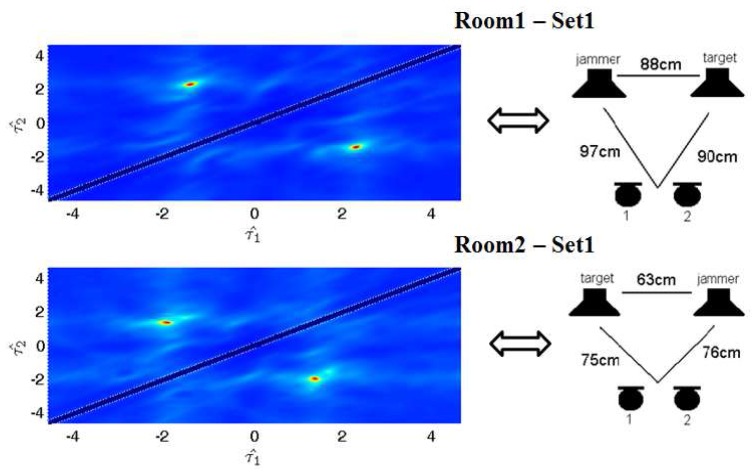
ITD estimation results and experimental set-up in room1 and room2. The horizontal coordinate corresponds to $\hat{\tau_{1}}$, and the vertical coordinate corresponds to $\hat{\tau_{2}}$. The distance between two microphones is 8 cm.

The data of D2-2 sets of the Signal Separation Evaluation Campaign (SiSEC) [26] consists of two-microphone real world recordings. We applied the proposed method to set1 for both room1 and room2. We only compare our method with the classical Fast-ICA [27], since the results with other methods can be found online. Figure 9 shows ITD estimation details. Table 1 and Table 2 illustrate that our method can achieve competitive results.

**Figure 10:**
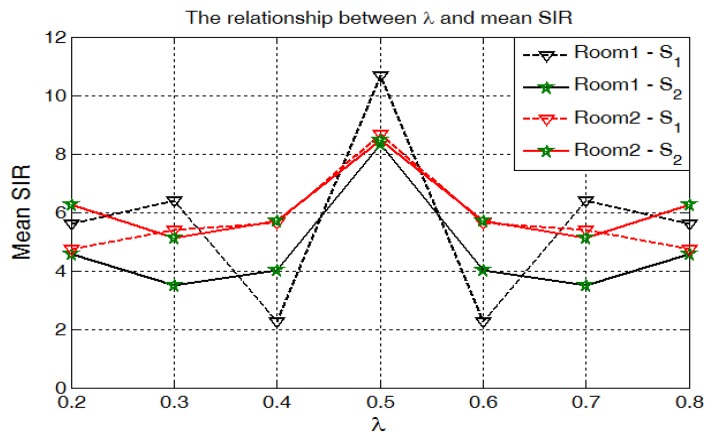
Average Signal-to-Interference Ratio (SIR) with different λ. We calculate the mean of SIR for each λ. The result demonstrates that λ=0.5 provides the best performance, which is identical to our theoretical analysis. Furthermore, separation results are symmetrical to λ when we adopt the signal-to-noise ratio based on $Y_{1}\left[m,k\right]$ and $Y_{2}\left[m,k\right]$ to generate the ideal binary mask.

In Figure 10, we demonstrate the trends between λ and mean SIR for room1 and room2. Mean SIR is symmetrical to λ=0.5, where mean SIR achieves the best performance. These characteristics are consistent with our method.

Table 4 shows the separation performance for both room1 and room2.

## 6. Conclusions

In this paper, we have proposed a novel method based on DSB for dual-channel sources separation. Our method, for the first time, employs the extension of DSB for estimating interaural time difference (ITD) and illustrates the connection between ideal binary mask and DSB amplitude ratio. Our method is valid for phase wrap-around. Although our method is based on the assumption of an anechoic environment, the results illustrate the effectiveness for low reverberation environment ($RT_{60}$ = 150 ms). Objective evaluations demonstrate the effectiveness of our proposed methods.

In this paper, we focus on the estimation of the interaural time differences (ITD). In fact, the construction of an effective masking model is also very critical. We could attempt to replace our Time-Frequency Masking with an NMF2D model as proposed in [14], and adopt the GEM-MU and Gamma-Exponential process to separate sound sources. Moreover, in the presence of background noise, the idea of noise reduction in [3] is also valuable for our dual-channel speech separation.

## Figures and Tables

**Table 1:** ITD estimation on $S_{1}S_{2}^{1}$.

Anechoic			${\mathrm{RT}}_{60}$ = 150 ms		
Method	$S_{1}$	$S_{2}^{1}$	Method	$S_{1}$	$S_{2}^{1}$
Real ITD	0.000	2.373	Real ITD	0.000	2.373
DUET	0.058	2.370	DUET	0.520	2.560
Phat	0.017	2.502	Phat	0.217	2.500
Izumi	0.093	2.502	Izumi	0.337	2.946
Proposed	0.024	2.402	Proposed	0.179	2.428

**Table 2:** Interaural Time Difference (ITD) estimation on $S_{1}S_{2}^{2}$.

Anechoic			${\mathrm{RT}}_{60}$ = 150 ms		
Method	$S_{1}$	$S_{2}^{2}$	Method	$S_{1}$	$S_{2}^{2}$
Real ITD	0.000	4.060	Real ITD	0.000	4.060
DUET	0.020	3.963	DUET	1.844	3.448
Phat	0.055	4.009	Phat	0.117	4.122
Izumi	0.045	4.018	Izumi	0.043	4.067
Proposed	0.012	4.039	Proposed	0.042	4.045

**Table 3:** ITD estimation on $RT_{60}$ = 150 ms with different microphone distances.

Mic-Distance	5 cm		10 cm		15 cm	
Method	$S_{1}$	$S_{2}^{1}$	$S_{1}$	$S_{2}^{1}$	$S_{1}$	$S_{2}^{1}$
Real ITD	0.000	1.187	0.000	2.373	0.000	3.560
DUET	0.271	1.069	0.520	2.560	1.678	3.135
PHAT	0.163	1.296	0.217	2.500	0.126	3.652
Izumi	0.234	1.334	0.337	2.946	0.031	3.891
Proposed	0.112	1.125	0.179	2.428	0.041	3.527

**Table 4:** Signal-to-Interference Ratio (SIR) evaluations based on room1 and room2.

**Room1**		**x1**	**x2**	**x3**	**x4**	**x5**	**x6**
Proposed	$S_{1}$	11.8	7.8	14.7	26.4	4.9	−0.9
	$S_{2}$	10.5	12.2	−9.2	2.7	14.0	21.2
ICA	$S_{1}$	0.3	−1.3	10.2	18.6	−2.6	−7.8
	$S_{2}$	3.3	4.8	−8.34	−7.6	10.0	18.3
**Room2**		**x1**	**x2**	**x3**	**x4**	**x5**	**x6**
Proposed	$S_{1}$	3.3	6.2	12.3	27.5	3.2	1.0
	$S_{2}$	12.8	11.1	−10.0	−1.3	15.8	22.5
ICA	$S_{1}$	−3.2	−1.3	6.6	19.6	−4.3	−9.1
	$S_{2}$	6.2	4.8	−7.3	−8.5	12.0	19.4

^1^ The definition of ICA is “Independent Component Analysis”.
